# The association between shift work and chronic kidney disease in manual labor workers using data from the Korea National Health and Nutrition Examination Survey (KNHANES 2011–2014)

**DOI:** 10.1186/s40557-018-0279-z

**Published:** 2018-12-14

**Authors:** Jun Young Uhm, Hyoung-Ryoul Kim, Gu Hyeok Kang, Young Gon Choi, Tae Hwi Park, Soo Young Kim, Seong Sil Chang, Won Oh Choo

**Affiliations:** 1 Department of Occupational & Environmental Medicine, Eulji University Hospital, Daejeon, Republic of Korea 35233; 2 Department of Occupational and Environmental Medicine, Seoul St. Mary’s Hospital, College of Medicine, The Catholic University of Korea, 222 Banpo-daero, Seocho-gu, Seoul, 137701 Republic of Korea

**Keywords:** Shift work, Chronic kidney disease, Circadian disruption, KNHANES

## Abstract

**Objective:**

Kidneys are organs having a biological clock, and it is well known that the disruption of the circadian rhythm increases the risk of chronic kidney disease (CKD), including the decline of renal and proteinuria. Because shift work causes circadian disruption, it can directly or indirectly affect the incidence of chronic kidney disease. Therefore, the purpose of this study was to investigate the association between shift work and chronic kidney disease using a Korean representative survey dataset.

**Methods:**

This study was comprised of 3504 manual labor workers over 20 years of age from data from the fifth and sixth Korea National Health and Nutrition Examination Survey (2011–2014). The work schedules were classified into two types: day work and shift work. The estimated glomerular filtration rate, which is the ideal marker of renal function, was estimated according to the Chronic Kidney Disease Epidemiology Collaboration creatinine equation, and chronic kidney disease was defined as urinary albumin to a creatinine ratio equal to or high than 30 mg/g and/or estimated glomerular filtration rate lower than 60 mL/min/1.73 m^2^. The cross-tabulation analysis and multivariate logistic regression analysis were performed to confirm the association between shift work and chronic kidney disease stratified by gender.

**Results:**

The risk of CKD showed a significant increase (odds ratio = 2.04, 95% confidence interval = 1.22, 3.41) in the female worker group. The same results were obtained after all confounding variables were adjusted (odds ratio = 2.34, 95% confidence interval = 1.35, 4.07). However, the results of the male worker group were not significant.

**Conclusions:**

In this study using nationally representative surveys, we found that the risk of CKD was higher female workers and shift work. Future prospective cohort studies will be needed to clarify the causal relationship between shift work and CKD.

## Background

Since the beginning of the second industrial revolution, people have been able to illuminate the night as bright as the day from the advent of electricity and technology. This phenomenon led to an environment in which employees were allowed to work at night, and the systematization and efficiency of the work has increased [1]. In addition, as industries diversify and globalize, more work must be completed in a shorter time, and there is an increasing number of jobs that must be performed around the clock [2]. Based on this trend, shift work, a system that increases the total work time of a company by distributing workers into groups working at different times, has been introduced [3].

Although there are some differences in literature, shift work is defined as all types of work other than those at the regular working time (7 a.m.- 6 p.m.), according to the National Institute for Occupational Safety and Health (NIOSH) [4]. In industrialized countries, including the United States, at least 15% of workers are working under these conditions, and the proportion is gradually rising despite constant reporting of the detrimental effects of shift work on the human body [5, 6].

Almost all organisms, including humans, have a biological clock [7]. Numerous circadian rhythms were identified according to species, and humans showed a diurnal preference, which is the tendency towards morning or evening [8]. Since organisms follow such characteristics from the cellular level to organs, disruption to the circadian rhythm can lead to adverse health outcomes from several pathways [9]. Shift work inevitably causes circadian disruption [10], and several studies found evidence that such working conditions increased the risk of various diseases. First, shift work is known to increase the risk of cardiovascular disease (CVD), such as coronary heart disease (CHD), as well as hypertension and type 2 diabetes [11]. In addition, the International Agency for Research on Cancer (IARC) classified shift work as a ‘probable human carcinogen 2A’ [12], and some studies reported that the risk of breast cancer in female shift workers was relatively higher than that in female regular day workers, while the risk of prostate cancer in male shift workers was relatively higher than that in male regular day workers [13, 14]. In addition, shift work was reported to increase the risk of mental disorders, such as anxiety and depressive disorders [15].

Chronic kidney disease (CKD) is on the rise worldwide, and is an important public health issue because it increases patient mortality and burdens those affected with medical expenses [16]. The kidney is an organ with a biological clock, so the disruption of circadian rhythm by disturbed sleep may induce over-activation of the renin-angiotensin-aldosterone system (RAAS) and oxidative stress, which are known to increase the risk of CKD by compromised renal function or kidney damage [17–19]. Since shift work causes this circadian disruption [10], it may directly or indirectly affect the occurrence of CKD.

Some previous studies suggested a relationship between shift work and CKD. However, these studies were conducted on workers at specific workplaces, and there are few large-scale population-based epidemiological studies. Therefore, this study was conducted to analyze the association between shift work and CKD using the nationwide population-based survey, the Korean National Health and Nutrition Examination Survey (KNHANES).

## Materials and methods

### Subjects

**Fig. 1:**
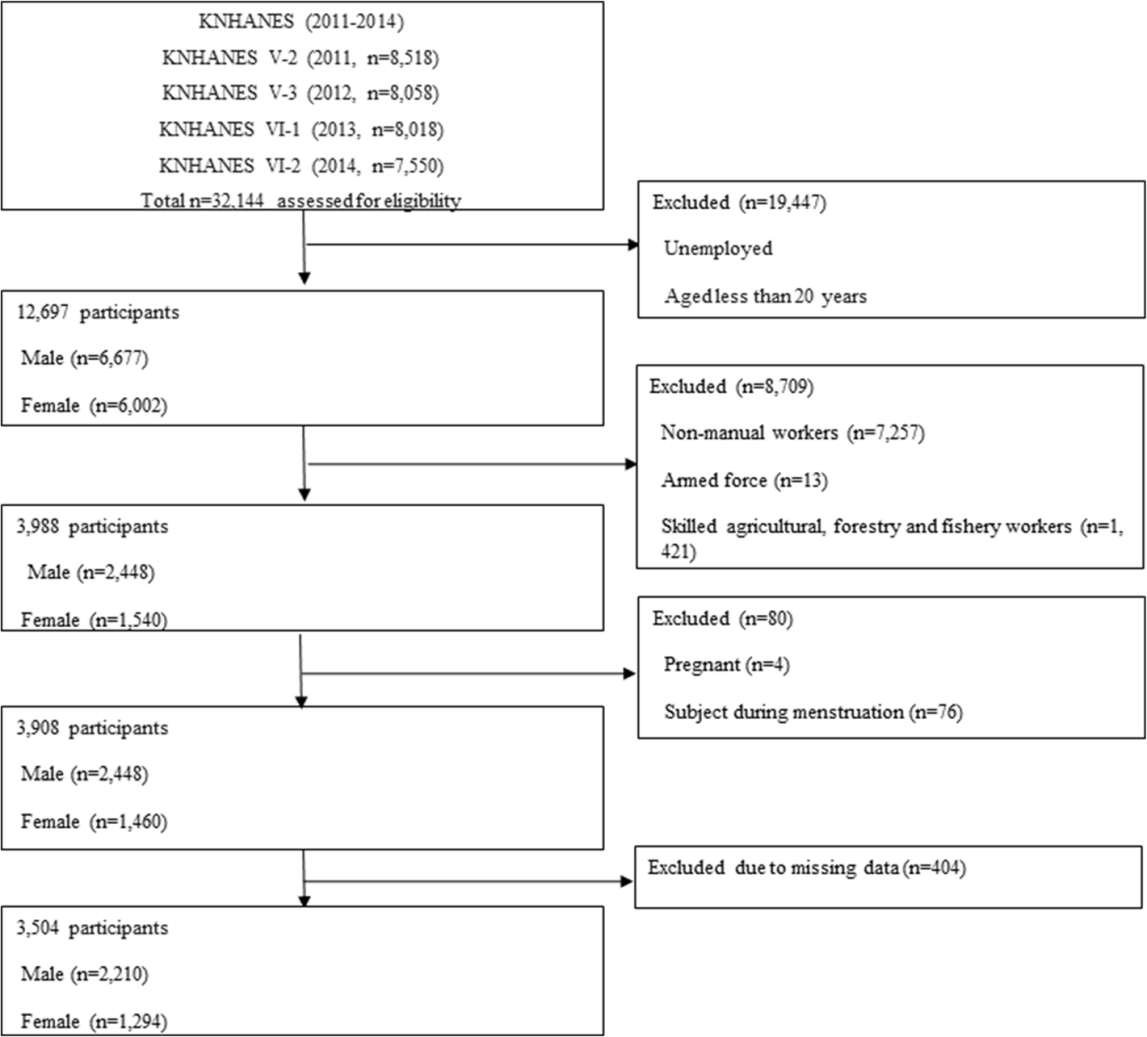
Flow chart of inclusion and exclusion of the study participants. KNHANES: Korean National Health and Nutritional Examination Survey

The second (2011) and the third year (2012) data of the fifth Korean National Health and Nutrients Examination Survey (KNHANES) (2010–2012), and the first (2013) and the second year (2014) data of the sixth KNHANES (2013–2015) were used in this study. KNHANES is a national cross-sectional survey collected annually by the governmental organization, Korea Centers for Disease Control and Prevention (KCDC), and is provided as secondary data designed according to multistage stratified and cluster sampling. The data, which was used to assess the health and nutritional status of the Korean population, included variables such as age, gender, serum and urinary creatinine levels, and urinary albumin levels required to identify the association between shift work and CKD. It also included several demographic variables and clinical characteristics known to affect renal function and proteinuria. The occupations of the subjects in KNHANES were classified into ten occupational categories according to the KSCO standard, which was based on the International Standard Classification of Occupations (ISCO-08) of International Labor Organization (ILO), and which presented the criteria of classification for job related study by classifying and collecting job information consistently through statistical survey or census [20]. After excluding the armed forces, the remaining workers were divided into two occupational types (manual labor workers and non-manual labor workers) according to the degree of physical strain. The manual labor worker group consisted of skilled agricultural, forestry and fishery workers, craft and related trades workers, equipment, machine operating and assembling workers, and elementary workers, whereas the non-manual labor worker group consisted of managers, professionals and related workers, clerks, service workers and sales workers. The total number of participants in the four-year period of KNHANES was 32,144, of whom 12,679 were over 20 years old and were employed. Among these 12,679 workers, 3988 were selected, excluding the non-manual labor workers (*n* = 7257), armed forces (*n* = 13), and skilled agricultural, forestry and fishery workers (*n* = 1421). Among these, a total of 3504 participants were finally selected as the subjects of this study after excluding the pregnant workers (*n* = 4), and female workers during their menstrual cycle (*n* = 76), since their urine albuminuria levels could be measured incorrectly, and those with missing data (*n* = 612) in sequence (Fig. 1).

### Assessment of day work and shift work

The National Institute for Occupational Safety and Health (NIOSH) defined shift work as all types of work conditions except for regular day work hours [7 a.m. to 6 p.m.] [4]. In this study, however, regular day work hours were set from 6 a.m. to 6 p.m., and the remaining work hours were set as shift work time (fixed evening shift work [2 p.m. to midnight], fixed night shift work [9 p.m. to 8 a.m.], regular day-night shift work, 24-h rotating shift work, split shift work, and irregular shift work) according to the work schedule items in KNHANES questionnaire.

### Assessment of renal function and definition of chronic kidney disease

Since the glomerular filtration rate (GFR) is known as the ideal marker that best reflects renal function, it was set as an indicator of renal function levels in this study [21, 22]. CKD was defined as the glomerular filtration rate less than 60 mL/min/1.73 m^2^ (less than 50% of normal renal function) and/or urinary albumin to creatinine ratio (UACR) 30 mg/g or more according to the Kidney Disease Improving Global Outcomes (KDIGO) criteria. [23]. GFR was estimated using the CKD-EPI (Chronic Kidney Disease Epidemiology Collaboration) creatinine equation. The CKD-EPI creatinine equation is eGFR = 141 x min(Scr/κ,1)^α^ x max(Scr/κ,1)^-1.209^ × 0.993^age^ × 1.018 [if female] × 1.159 (if black), and the abbreviation and unit of variables included in the equation are eGFR (estimated glomerular filtration rate, mL/min/1.73 m^2^), Scr (serum creatinine, mg/dL), age (years), κ = 0.7 (female) or 0.9 (male), α = − 0.329 (female) or − 0.411 (male), min = the minimum of Scr/κ or 1, and max = the maximum of Scr/κ or 1. Since KNHANES only targets the Korean population, variables related to the race were not considered in this equation. The urinary albumin to creatinine ratio (UACR, mg/g) was obtained by dividing the urinary albumin concentration by the urinary creatinine concentration. Blood sampling was performed in subjects that fasted for at least 8 h to obtain serum creatinine levels, and each sample was measured by a Jaffe rate-blanked and compensated method (Hitachi Automatic Analyzer 7600–210, Hitachi, Tokyo, Japan) in a professional blood test laboratory. Urinary albumin levels and urinary creatinine levels were collected from random urine in the first urine of the morning and measured by a turbidimetric assay (Hitachi Automatic Analyzer 7600) and Jaffe rate-blanked and compensated method (Hitachi Automatic Analyzer 7600–210, Hitachi, Tokyo, Japan), respectively.

### Potential covariates

Factors known to affect CKD from previous studies were included as potential covariates in the study [24, 25]. Demographic data such as age, sex, education level, household income, smoking status, and alcohol intake and work conditions such as work hours per week were collected via a standardized questionnaire. Work hours per week were classified as less than 48 h and over 48 h according to the ILO definition of long working hours [26]. Education levels were divided into elementary graduates or below, middle school graduates, high school graduates, and college graduates or higher. Household income was divided into low quarters, low-middle, middle-high, and high-levels. Smoking status was classified as a non-smoker, ex-smoker, and current smoker. Alcohol intake was classified as never, less than once per month, twice or three times per month, and more than 4 times per month according to the frequency of drinking alcohol per month in the previous year. To obtain clinical data, a trained examiner measured blood pressure and anthropometric data, such as the subject’s height and weight, and obtained fasting glucose and total cholesterol levels by blood sampling. After measuring height and body weight by standardized techniques, the body mass index (BMI) was calculated by dividing the body weight in kilograms by the height in meters squared (kg/m^2^). Then, BMI was categorized as underweight if lower than 18.5 kg/m^2^, normal if between 18.5 kg/m^2^ and 24.99 kg/m^2^, overweight if between 25 kg/m^2^ and 29.99 kg/m^2^, and obese if higher than 30 kg/m^2^ according to the obesity criteria of Centers for Disease Control and Prevention (CDC) [27]. Blood pressure (BP) was measured with a mercury sphygmomanometer (Baumanometer Wall Unit 33, Baum, Copiague, NY, USA) after a 5-min stable period by sitting. Systolic blood pressure (SBP) and diastolic blood pressure (DBP) were measured three times, and the mean value of the second and third BPs was used for analysis. Hypertension was defined as SBP ≥ 140 mmHg, DBP ≥ 90 mmHg, or taking the antihypertensive agent for 20 days or more per month according to the 7th Joint National Committee on prevention, detection, evaluation, and treatment of high blood pressure (JNC-7) guidelines [28]. Blood sampling was performed in the morning after fasting for at least 8 h. With each blood sample, total cholesterol was analyzed by an enzymatic method (Hitachi Automatic Analyzer 7600–210, Hitachi, Tokyo, Japan), and fasting glucose was analyzed by hexokinase UV (Hitachi Automatic Analyzer 7600–210, Hitachi, Tokyo, Japan). Diabetes mellitus was defined as fasting glucose ≥126 mg/dL, from a previous diagnosis by a physician, or when taking diabetes treatment by medication or injecting insulin.

### Statistical analyses

A weighted complex sample analysis was used for data analysis of 3504 subjects taking account of the characteristics of the data used in the study. After the data were stratified by gender, chi-square tests were performed to examine the distributions of categorical variables, and Student’s *t*-tests were performed to compare means of continuous variables. Subsequently, both male and female subjects were classified into day worker group and shift worker group respectively to perform Student’s *t*-tests for continuous variables and chi-squared tests for categorical variables to confirm differences in general characteristics by work schedule. To determine if there was a significant association between CKD and various variables including work schedule, complex samples chi-squared tests were performed, and odds ratios (OR) were calculated by complex samples logistic regression analysis. To reflect the impact of each variable, age, BMI, SBP, total cholesterol, fasting glucose, diabetes mellitus and hypertension were adjusted in model 1, and work hours per week, household income, education level, smoking, and alcohol intake were additionally adjusted in model 2. Statistical significance was defined as *p* < 0.05 and SPSS version 18.0 (SPSS Inc., Chicago, IL, USA) was used for the statistical analysis.

## Results

### Demographic and clinical characteristics of the subjects

The detailed demographic and clinical characteristics of the subjects are presented in Tables 1 and 2. The total number of subjects was 3504, with 2210 men (63.1%) and 1294 women (36.9%). The mean age of the subjects was 47.9 years, and the mean age of the male and female subjects was 46.1 years and 52.2 years, respectively. In terms of socioeconomic factors, the proportion of men with higher education levels and higher household incomes were significantly higher than those of women. Among lifestyle factors, the proportion of smokers and the subjects drinking more than twice per month were higher in males. In terms of work conditions, the proportion of working less than 48 h per week was larger than that of working over 48 h per week in both sexes, and the proportion of working over 48 h per week was significantly higher in male than in females. The prevalence of CKD was significantly higher in males who worked less than 48 h per week than in males who worked over 48 h per week, but there was no significant difference in females.

**Table 1:** Demographic and clinical characteristics of the subjects

Variable	Male	Female	Total	*p*-value^a^
Total	2210	1294	3504	
Age (years)	46.1 ± 0.3	52.2 ± 0.5	47.9 ± 0.3	< 0.001
20–29	133 (10.7)	41 (6.1)	174 (9.4)	< 0.001
30–39	397 (21.8)	103 (9.3)	500 (18.2)	
40–49	466 (26.1)	265 (26.1)	731 (26.1)	
50–59	628 (27.2)	406 (31.4)	1034 (28.4)	
≥ 60	586 (14.2)	479 (27.1)	1065 (17.9)	
Education				< 0.001
≤ Elementary school	417 (14.2)	577 (37.4)	994 (20.9)	
Middle school	393 (16.0)	215 (17.1)	608 (16.3)	
High school	1006 (50.5)	428 (39.3)	1434 (47.3)	
≥ College or graduate school	394 (19.3)	74 (6.3)	468 (15.5)	
Household income				< 0.001
Low	234 (7.6)	336 (22.1)	570 (11.8)	
Low-middle	731 (33.6)	411 (31.5)	1142 (33.0)	
Middle-high	741 (35.1)	333 (28.8)	1074 (33.3)	
High	504 (23.7)	214 (17.6)	718 (21.9)	
Smoking status				< 0.001
Non-smoker	369 (17.2)	1163 (87.4)	1532 (37.5)	
Ex-smoker	822 (33.0)	50 (4.7)	872 (24.8)	
Current smoker	1019 (49.8)	81 (8.0)	1100 (37.7)	
Alcohol intake (per month)				< 0.001
Never	297 (11.6)	507 (35.8)	804 (18.6)	
≤ 1	420 (19.2)	476 (37.3)	896 (24.4)	
2–3	564 (27.1)	210 (18.1)	774 (24.5)	
≥4	929 (42.2)	101 (8.8)	1030 (32.5)	
Work hours per week				
≤ 48	1191 (52.3)	1019 (76.4)	2210 (59.3)	< 0.001
> 48	1019 (47.7)	275 (23.6)	1294 (40.7)	
Work schedule				< 0.001
Day work	1707 (79.9)	1133 (86.4)	2840 (81.7)	
Shift work	503 (20.1)	161 (13.6)	664 (18.3)	
CKD^b^				0.013
No	2023 (93.2)	1158 (90.5)	3181 (92.4)	
Yes	187 (6.8)	136 (9.5)	323 (7.6)	
BMI (kg/m^2^)				0.098
Underweight (< 18.5)	48 (2.4)	37 (3.2)	85 (2.6)	
Normal (18.5–25)	1355 (61.3)	808 (62.1)	2163 (61.5)	
Overweight (25–30)	730 (32.4)	378 (29.2)	1108 (31.5)	
Obese (≥30)	77 (4.0)	71 (5.5)	148 (4.4)	
Diabetes mellitus				0.058
No	1935 (89.6)	1165 (91.7)	3100 (90.2)	
Yes	275 (10.4)	129 (8.3)	404 (9.8)	
Hypertension				0.885
No	1461 (70.8)	858 (71.1)	2319 (70.9)	
Yes	749 (29.2)	436 (28.9)	1185 (29.1)	
SBP (mmHg)	120.1 ± 0.39	119.6 ± 0.64	120.0 ± 0.33	< 0.001
DBP (mmHg)	79.3 ± 0.30	75.2 ± 0.35	78.1 ± 0.24	< 0.001
Fasting glucose (mg/dl)	100.5 ± 0.54	98.0 ± 0.64	99.8 ± 0.42	< 0.001
Total cholesterol (mg/dl)	189.0 ± 0.86	194.7 ± 1.28	190.6 ± 0.71	< 0.001

BMI, body mass index; CKD, chronic kidney disease; CKD-EPI, CKD epidemiology collaboration, DBP, diastolic blood pressure; eGFR, estimated glomerular filtration rate; SBP, systolic blood pressure; UACR, urinary albumin-to-creatinine ratio

^a^calculated by complex samples, chi-squared test for categorical variables and t-test for continuous variables

^b^defined as UACR> 30 mg/g and/or eGFR< 60 mL/min/1.73 m2 estimated by the CKD-EPI equation

Values are expressed as unweighted counts and estimated percentages N(%) for categorical variables and as mean ± standard errors for continuous variables

**Table 2:** Demographic and clinical characteristics of subjects by work schedule

Variable	Male		Female	
	Day work	Shift work	Day work	Shift work
Total	1707 (79.9)	503 (20.1)	1133 (86.4)	161 (13.6)
Age (years) ^a,b^				
20–29	108 (11.1)	25 (9.3)	33 (5.5)	8 (10.1)
30–39	312 (22.2)	85 (20.1)	93 (9.9)	10 (5.4)
40–49	376 (26.9)	90 (22.9)	233 (26.5)	32 (23.7)
50–59	503 (27.6)	125 (25.8)	346 (30.4)	60 (38.1)
≥60	408 (12.2)	178 (21.9)	428 (27.7)	51 (22.8)
Education^a^				
≤ Elementary school	308 (13.7)	109 (16.5)	513 (38.0)	64 (33.4)
Middle school	321 (17.0)	72 (11.9)	191 (17.5)	24 (14.5)
High school	788 (51.0)	218 (48.4)	366 (38.4)	62 (44.8)
≥ College or graduate school	290 (18.3)	104 (23.2)	63 (6.1)	11 (7.3)
Household income				
Low	190 (7.5)	44 (7.9)	296 (22.2)	40 (21.4)
Low-middle	569 (34.3)	162 (30.7)	366 (32.0)	45 (28.7)
Middle-high	568 (35.3)	173 (34.4)	289 (28.6)	44 (29.8)
High	380 (22.9)	124 (27.0)	182 (17.2)	32 (20.0)
Smoking status				
Non-smoker	279 (17.3)	90 (16.7)	1025 (88.1)	138 (83.0)
Ex-smoker	607 (31.6)	215 (38.3)	43 (4.7)	7 (4.4)
Current smoker	821 (51.1)	198 (45.0)	65 (7.2)	16 (12.7)
Alcohol intake (per month)				
Never	216 (10.8)	81 (14.6)	445 (35.9)	62 (34.8)
≤ 1	314 (19.0)	106 (19.9)	421 (37.7)	55 (35.0)
2–3	425 (26.8)	139 (28.1)	183 (18.2)	27 (17.2)
≥4	752 (43.4)	177 (37.4)	84 (8.2)	17 (12.9)
Work hours per week ^a,b^				
≤ 48	1017 (55.8)	174 (38.5)	898 (77.5)	121 (69.2)
> 48	690 (44.2)	329 (61.5)	235 (22.5)	40 (30.8)
CKD ^b^				
No	1565 (93.2)	458 (93.1)	1024 (91.5)	134 (84.0)
Yes	142 (6.8)	45 (6.9)	109 (8.5)	27 (16.0)
BMI (kg/m^2^)				
Underweight (< 18.5)	40 (2.6)	8 (1.6)	30 (2.7)	7 (6.2)
Normal (18.5–25)	1049 (61.3)	306 (61.1)	709 (62.3)	99 (61.1)
Overweight (25–30)	553 (31.8)	177 (34.8)	336 (29.8)	42 (24.9)
Obese (≥30)	65 (4.3)	12 (2.6)	58 (5.1)	13 (7.8)
Diabetes mellitus				
No	1500 (90.1)	458 (87.6)	1020 (91.9)	145 (90.8)
Yes	207 (9.9)	45 (12.4)	113 (8.1)	16 (9.2)
Hypertension				
No	1119 (70.7)	342 (71.3)	742 (70.4)	116 (75.3)
Yes	588 (29.3)	161 (28.7)	391 (29.6)	45 (24.7)
SBP (mmHg)^a,b^	120.3 ± 0.46	119.5 ± 0.68	119.9 ± 0.67	117.3 ± 1.39
DBP (mmHg) ^a,b^	79.5 ± 0.32	78.7 ± 0.65	75.4 ± 0.37	73.7 ± 0.76
Fasting glucose (mg/dl)^a,b^	100.8 ± 0.64	99.1 ± 0.95	98.0 ± 0.62	98.0 ± 2.33
Total cholesterol (mg/dl)^a,b^	189.1 ± 0.98	188.5 ± 1.78	195.7 ± 1.36	188.8 ± 3.50

Values are expressed as unweighted counts and estimated percentages N(%) for categorical variables and as the mean ± standard error for continuous variables. ^a^Significant differences between day work and shift work in male subjects (*p* < 0.05), ^b^Significant differences between day work and shift work in female subjects (*p* < 0.05)

In terms of work schedule, the proportion of regular day workers was higher than that of shift workers in both males and females, and the proportion of day workers in females was significantly higher than that in males. BMI was not significantly different between males and females, and there was no significant gender difference in the incidence of diabetes mellitus and hypertension. SBP, DBP, total cholesterol, and fasting glucose were significantly higher in males, while CKD prevalence was significantly higher in females (Table 1). Age, education levels, work hours per week, SBP, DBP, total cholesterol levels and fasting glucose levels were significantly different between the group of day workers and shift workers in males, whereas age, work hours per week, SBP, DBP, total cholesterol level, fasting glucose level, and CKD prevalence were significantly different between the group of day workers and shift workers in females (Table 2).

### Prevalence of CKD according to work schedules and associated factors

The subjects were classified into two groups (the non-CKD and the CKD) according to the 2012 KDIGO guideline for CKD evaluation, and the prevalence of CKD according to each factor was investigated. The prevalence of CKD tended to increase with age in males, which tended to be similar in females except for females in their 20s. As to socioeconomic factors, the prevalence of CKD tended to be significantly increased with lower levels of education in both sexes. In addition, the prevalence of CKD tended to increase as the household income level decreased in males, and it showed a similar tendency in females, except in high-levels of a household level. For work conditions, the prevalence of CKD was significantly higher in males who worked less than 48 h per week than in males who have worked more than 48 h per week, but there was no significant difference in females. There was no significant difference in the prevalence between shift workers and day workers in males, whereas the prevalence was significantly higher in shift workers than in day workers in females. For clinical characteristics, the prevalence of CKD was significantly higher in subjects with diabetes mellitus and hypertension than in subjects without the diseases regardless of gender. There were no statistically significant differences in the prevalence of CKD according to smoking status, alcohol intake and BMI in both sexes (Table 3).

**Table 3:** Prevalence of CKD according to work schedules and associated variables in subjects

Variable	Male			Female		
	Non CKD	CKD ^a^	*p*-value^b^	Non CKD	CKD ^a^	*p*-value^b^
Total	2023 (93.2)	187 (6.8)		1158 (90.5)	136 (9.5)	
Age (years) ^a,b^			< 0.001			< 0.001
20–29	131 (98.3)	2 (1.7)		37 (91.6)	4 (8.4)	
30–39	380 (95.9)	17 (4.1)		101 (96.6)	2 (3.4)	
40–49	441 (95.1)	25 (4.9)		252 (95.6)	13 (4.4)	
50–59	557 (91.8)	51 (8.2)		370 (90.9)	36 (9.1)	
≥60	494 (84.1)	92 (15.9)		398 (82.6)	81 (17.4)	
Education^a^			< 0.001			< 0.001
≤ Elementary school	357 (87.1)	60 (12.9)		492 (85.4)	85 (14.6)	
Middle school	362 (93.7)	31 (6.3)		194 (91.6)	21 (8.4)	
High school	928 (93.5)	78 (6.5)		402 (94.1)	26 (5.9)	
≥ College or graduate school	376 (96.4)	18 (3.6)		70 (95.3)	4 (4.7)	
Household income			0.022			0.006
Low	193 (87.1)	41 (12.9)		281 (84.3)	55 (15.7)	
Low-middle	670 (93.8)	61 (6.2)		371 (91.4)	40 (8.6)	
Middle-high	686 (93.3)	55 (6.7)		310 (93.5)	23 (6.5)	
High	474 (94.0)	30 (6.0)		196 (91.6)	18 (8.4)	
Smoking status			0.931			0.902
Non-smoker	335 (93.1)	34 (6.9)		1040 (90.4)	123 (9.6)	
Ex-smoker	744 (92.9)	78 (7.1)		46 (92.5)	4 (7.5)	
Current smoker	944 (93.4)	75 (6.6)		72 (90.1)	9 (9.9)	
Alcohol intake (per month)^a^			0.453			0.093
Never	262 (92.5)	35 (7.5)		440 (87.8)	67 (12.2)	
≤ 1	392 (94.7)	28 (5.3)		432 (92.2)	44 (7.8)	
2–3	528 (93.7)	36 (6.3)		196 (93.6)	14 (6.4)	
≥4	841 (92.3)	88 (7.7)		90 (87.5)	11 (12.5)	
Work hours per week						
≤ 48	1067 (91.6)	124 (8.4)	0.004	911 (90.7)	108 (9.3)	0.680
> 48	956 (94.9)	63 (5.1)		247 (89.7)	28 (10.3)	
Work schedule			0.926			0.006
Day work	1565 (93.2)	142 (6.8)		1024 (91.5)	109 (8.5)	
Shift work	458 (93.1)	45 (6.9)		134 (84.0)	27 (16.0)	
BMI (kg/m^2^)			0.035			0.083
Underweight (< 18.5)	45 (95.6)	3 (4.4)		32 (86.0)	5 (14.0)	
Normal (18.5–25)	1260 (94.4)	95 (5.6)		738 (92.3)	70 (7.7)	
Overweight (25–30)	651 (91.2)	79 (8.8)		330 (88.2)	48 (11.8)	
Obese (≥30)	67 (88.4)	10 (11.6)		58 (84.3)	13 (15.7)	
Diabetes mellitus			< 0.001			< 0.001
No	1818 (95.0)	117 (5.0)		1065 (92.2)	100 (7.8)	
Yes	205 (77.1)	70 (22.9)		93 (71.7)	36 (28.3)	
Hypertension			< 0.001			< 0.001
No	1401 (96.4)	60 (3.6)		812 (94.7)	46 (5.3)	
Yes	622 (85.3)	127 (14.7)		346 (80.2)	90 (19.8)	

Values are expressed as unweighted counts and estimated percentages N (%)

^a^defined as UACR> 30 mg/g and/or eGFR< 60 mL/min/1.73 m2 estimated by the CKD-EPI equation

^b^calculated by complex samples and the chi-squared test

### Odds ratios of CKD according to work schedules

Table 4 shows the results of multiple logistic regression analyses conducted for male and female subjects, and displays the crude and adjusted odds ratios of CKD according to work schedules. As with the male workers, the OR of CKD for the group of shift workers compared to the group of day workers was not significant with OR 1.02 (95% CI = 0.66, 1.58). The OR was not significant with OR 0.97 (95% CI = 0.62, 1.53) after adjusting for age and clinical characteristics such as BMI, systolic blood pressure, total cholesterol level, fasting glucose level, history of diabetes mellitus and history of hypertension, and intake the OR was not significant with OR = 1.06 (95% CI = 0.65, 1.73), even after additional adjusting for work conditions and demographic variables such as work hours per week, household income, education level, smoking, alcohol intake. On the other hand, for female workers, the OR of CKD for the shift worker group compared to the day worker group was significantly high with OR = 2.04 (95% CI = 1.22, 3.41). The OR was significantly high with 2.35 (95% CI = 1.36, 4.08) after adjusting for age and clinical characteristics such as BMI, systolic blood pressure, total cholesterol level, fasting glucose level, history of diabetes mellitus and history of hypertension as well. Similar results were obtained with OR = 2.34 (95% CI = 1.35, 4.07), even after additional adjusting for work conditions and demographic variables such as work hours per week, household income, education level, smoking and alcohol intake (Table 4).

**Table 4:** Crude and adjusted odds ratios for chronic kidney disease in male and female subjects

	Work schedule	Crude OR(95% CI)	Model 1^a^	Model 2^b^
Male	Day work	Reference	Reference	Reference
	Shift work	1.02 (0.66, 1.58)	0.97 (0.62, 1.53)	1.06 (0.65, 1.73)
Female	Day work	Reference	Reference	Reference
	Shift work	2.04 (1.22, 3.41)	2.35 (1.36, 4.08)	2.34 (1.35, 4.07)

OR, odds ratio; CI, confidence interval

^a^ Adjusted for age, body mass index, systolic blood pressure, total cholesterol, fasting glucose, diabetes mell*i*tus and hypertension

^b^ Adjusted for variables in model 1 plus work hours per week, household income, education level, smoking, and alcohol consumption

## Discussion

The goal of this study was to investigate the association between CKD and shift work in the growing workforce, and the results showed gender disparities in the association. Specifically, shift work was associated with CKD in the female worker group, whereas no significant association was found between shift work and CKD in the male worker group.

There is some evidence suggesting that shift work is associated with CKD. Boogaard et al. studied the relationship between albumin excretion and shift work [29]. The subjects in this study were divided into three groups: a group of shift workers from the organochlorine plants who were at risk of exposure to potentially nephrotoxic substances such as halogenated hydrocarbons, a group of shift workers and a group of day workers without any risk of occupational exposure to the substances. It was shown that regardless of exposure to nephrotoxic chemicals, the urinary albumin levels were high depending on shift worker groups, suggesting that the disruption of circadian rhythm may cause kidney damage. A cross-sectional study with 3000 manual workers by Kang et al. showed an association between shift work and microalbuminuria as a risk predictor of CVD, suggesting that a urine albumin test can be a practical method to predict and prevent CVD in shift workers who are more at risk of CVD than day workers [30]. Because microalbuminuria is not only a predictor of CVD, but also falls into the category of albuminuria, indicating kidney damage, it partially belongs to the diagnostic criteria for CKD. Therefore, it can be said that the study indirectly suggested the link between shift work and CKD. In a study with policemen to investigate the association between shift work and renal function, on the other hand, the mean eGFR was significantly lower in night shift worker group than in regular day worker group and was significantly lower as the percentage of hours worked at night shift increased. Thus, the study showed a night shift is associated with the decreased GFR [25].

The core pathophysiological mechanism among theoretical backgrounds that explain this association is the disruption of the circadian rhythm. Circadian rhythm, which is coordinated by the suprachiasmatic nucleus (SCN) of the hypothalamus, is one of the fundamental characteristics of most organisms [7], and it has a systemic effect on the human body from the level of cells to organs [31]. Therefore, the kidney may receive a deleterious effect due to the circadian disruption caused by shift work.

The kidney is an organ with a peripheral circadian clock; hence most of the renal physiologic processes, such as the regulation of the renin-angiotensin-aldosterone system (RAAS), follow the circadian rhythm. RAAS is well known to regulate the balance of fluids and electrolytes, and thus blood pressure in the body [18]. The circadian disruption by shift work, the over-activation of RAAS produces excessive angiotensin II, which causes vasoconstriction of efferent glomerular arterioles, results in increased intra-glomerular pressure, followed by ultrafiltration of plasma proteins [32]. In addition, increased activity of aldosterone can cause damage and remodeling of vascular endothelial cells, leading to protein leakage [33]. A glomerulus is a tuft of capillaries responsible for the filtration of blood components in the nephron of the kidney, so systemic vessel damage for certain reason can cause damage to the glomerulus as well [18, 32]. Therefore, it may cause proteinuria and decreased glomerular filtration rate, increasing the risk of CKD.

Meanwhile, shift work is known to induce oxidative stress [19]. Reactive oxygen species (ROS), due to their unpaired electrons, are highly reactive molecules. The state of overproducing ROS beyond an acceptable range of endogenous antioxidants is called oxidative stress. Oxidative stress reduces endothelial dysfunction by decreasing the bioavailability of NO, which plays a crucial role in smooth muscle relaxation [34]. Endothelial dysfunction is known to be one of the etiologies of DM and CVD, and these diseases are the leading causes of CKD [35, 36]. In addition, since the glomerulus is composed of capillaries with an endothelial lining, endothelial dysfunction may lead to reducing glomerular function [32, 37]. In other words, oxidative stress by shift work may induce endothelial dysfunction, which may directly or indirectly affect CKD development.

The link between shift work and CKD was partly explained by previous studies on sleep disturbance. McMullan et al. conducted an 11-year follow-up study with 4238 female participants, which showed that shorter sleep duration was independently associated with decreased renal function. [17]. Similarly, in a 5-year follow-up study of 431 patients with CKD conducted by Ricardo et al., a short sleep duration and poor sleep quality were found to increase the risk of ESRD, as well as the risk of proteinuria and renal function decline [38]. Since circadian disruption due to shift work may cause poor sleep conditions, these studies suggested that shift work may affect the development and progression of CKD [39].

According to the adjusted odds ratio for CKD, a significant relationship was found between shift work and CKD in the female worker, but not in the male worker group. The theoretical background of this gender disparity is not understood, but it can be explained by some hypotheses. In general, the load of domestic work, such as childcare and household chores is heavier in women than in men. This workload results in stress and decreased rest time, which may exert a synergistic effect on the development of CKD [40]. Furthermore, according to a systemic review as to differences in tolerance to shift work, males adapt better to shift work than females by maintaining a healthy lifestyle [41]. In terms of sleep quality, it is reported that female shift workers complained of difficulties falling asleep, and frequently take hypnotics and obtain deep sleep more than male shift workers [42, 43]. Therefore, differences in the lifestyle and sleep quality may have affected the gender differences in CKD prevalence.

Moreover, shift work is known to be associated with low socioeconomic status and lifestyle, as evidenced by dietary patterns and smoking [11, 44]. Smoking, low socioeconomic status and the increase in BMI due to changes in dietary patterns are all correlated with the development of CKD [35, 45]. Therefore, it can be assumed that the unhealthy lifestyle and low socioeconomic status related to shift workers might indirectly affect CKD prevalence. In our study, there were not any significant differences in lifestyle and socioeconomic status according to work schedules, other than the education levels among males. Thus, it is difficult to say that the difference in variables caused by shiftwork affected the prevalence of CKD in this study.

The global mean prevalence of CKD was found to be 13.4% in a meta-analysis of 42 CKD-related literature by Hill et al. [46]. On the other hand, the prevalence in this study was 7.6%, which was somewhat lower than the global prevalence. It was assumed that such disparities in prevalence may have been due to the presence of heterogeneity, such as differences in the inclusion criteria and demographic data of subjects [35]. However, in terms of gender difference, the prevalence of CKD was higher in females than in males (Male: 6.8%, Female: 9.5%) [46].

The blood pressure, fasting glucose, and total cholesterol were significantly lower in the shift group than in the normal group. There are two possible explanations, however, in regards to these results. First, on account of its statistic nature that the larger the study population, the smaller the standard deviation, a relatively small difference in the level of continuous variables can be interpreted as a significant difference in the level of these variables in a large population size. In this study, the number of the weighted study population is 6,469,033, which is relatively a large sample size. Thus, although the differences in the mean value of the blood pressure, fasting glucose, and total cholesterol level were less than 1 between day work group and shift work group, the results showed statistically significant differences between the two groups. Since the indices are very sensitive, the difference of 1 or less in these indices is not considered clinically significant. Therefore, although the values of these indices were significantly different statistically, they were not considered clinically different. Second, it is difficult to explain clearly how the adverse outcome between the CKD prevalence and the blood pressure occurred. However, because the blood pressure is affected by several factors (aging, smoking, alcohol intake, obesity, and the medical condition that can cause secondary hypertension) besides overactivation of RAAS by circadian rhythm disruption, hypertension can be caused by various factors. Moreover, the most common type of hypertension is primary hypertension, which comprises 95% of hypertension and has no identifiable cause. If a patient with hypertension took an antihypertensive medication a few hours before measuring the blood pressure, his or her blood pressure could be lowered, and this might alter the mean value. We believe that there is a limit to the nature of the survey items to see if there is a clinically meaningful difference in blood pressure between the two groups after adjusting all these factors.

### Limitation and strength

Some limitations hampered determination of the relatedness of shift work and CKD. First, the causal relationship between shift work and CKD was unclear because of the inborn limitation of the cross-sectional study. Second, because the demographic factors were collected based on the self-reported report by subjects, recall bias might have occurred. Third, the renal functional abnormalities and kidney damage should last more than 3 months in order to define CKD [23]. Since KNHANES was not conducted more than once a year, however, we could not confirm whether the decline in GFR or albuminuria persisted for more than 3 months. Finally, due to the limitations on the diversity of survey items, this study did not reflect the current use of medication that can affect kidneys and the history of kidney disorders (e.g. nephrotic syndrome, nephritis, polycystic kidneys, hydronephrosis, cortical scarring, and history of kidney transplantation).

Nevertheless, this study had several strengths that overcome these limitations. First, using the nationwide survey that represents the Korean population, we found an association between shift work and CKD. In addition, when estimating the renal function, a more accurate value of GFR could be obtained by applying the CKD-EPI creatinine equation, which is known to be more accurate than the MDRD equation. Finally, the diagnostic criteria for CKD were revised to define CKD based on not only the reduction of GFR, but also kidney damage, such as albuminuria, since 2012. [23]. This study was the first to define CKD by applying the new criteria to clarify the association with shiftwork. Based on these strengths, a complementary study will be needed after supplementing the above limitations.

## Conclusion

Because CKD increases mortality and has social and economic burdens, effort should be made at the public health level to lower the prevalence of CKD. This cross-sectional study using the nationally representative survey found that the risk of CKD was significantly higher in female shift workers than in female day workers. A well-designed cohort study should be undertaken to identify the causal relationship between shift work and CKD and to contribute to establishing a policy to improve the health and welfare of shift workers.

## Abbreviations

BMI: Body mass index
CI: Confidence interval
CKD: Chronic kidney disease
CKD-EPI: Chronic kidney disease epidemiology collaboration
CVD: Cardiovascular disease
eGFR: Estimated glomerular filtration rate
ESRD: End stage renal disease
ILO: International labor organization
KNHANES: Korea National Health and Nutrition Examination Surveys
MDRD: Modification of diet in renal disease
NIOSH: National Institute for Occupational Safety and Health
RAAS: Renin-angiotensin-aldosterone system
SCN: Suprachiasmatic nucleus
